# Simultaneous reduction and fixation using an anatomical suprapectineal quadrilateral surface plate through modified Stoppa approach in superomedially displaced acetabular fractures

**DOI:** 10.1038/s41598-022-19368-1

**Published:** 2022-09-08

**Authors:** Dae-Kyung Kwak, Seung-Hun Lee, Kang-Uk Lee, Ji-Hyo Hwang, Je-Hyun Yoo

**Affiliations:** 1 Department of Orthopaedic Surgery, Hallym University Sacred Heart Hospital, Hallym University College of Medicine, 22 Gwanpyeong-ro 170beon-gil, Dongan-gu, Anyang, 14068 South Korea; 2 Department of Orthopaedic Surgery, Kangnam Sacred Heart Hospital, Hallym University College of Medicine, South Korea Seoul,

**Keywords:** Outcomes research, Clinical trial design

## Abstract

Treatment of superomedially displaced acetabular fractures including a quadrilateral surface (QLS) is challenging. We present a surgical technique using an anatomical suprapectineal QLS plate through the modified Stoppa approach and report the availability of this plate to treat this fracture type along with the surgical outcomes. Sixteen consecutive patients (14 men and 2 women) who underwent surgical treatment using an anatomical suprapectineal QLS plate through a modified Stoppa approach for superomedially displaced acetabular fractures between June 2018 and June 2020, were enrolled retrospectively. These fractures included 11 both-column fractures and 5 anterior-column and posterior hemitransverse fractures, which were confirmed on preoperative 3-dimensional computed tomography. Surgical outcomes were clinically assessed using the Postel Merle d’Aubigné (PMA) score and visual analog scale (VAS) score at the final follow-up, and radiological evaluations were performed immediately after the operation and at the final follow-up. For comparative analysis, 23 patients who underwent internal fixation with the conventional reconstruction plate through modified ilioinguinal approach between February 2010 and May 2018, were selected. This control group was composed of 18 both-column fractures and 5 anterior-column and posterior hemitransverse fractures. The follow-up period was at least 1 year in all patients. The mean operation time and blood loss was 109 min, and 853 ml, respectively, whereas 236 min, and 1843 ml in control group. Anatomical reduction was achieved in 14 (87.5%) patients, while imperfect reduction was achieved in the remaining 2 patients. At the final follow-up, radiographic grades were excellent, fair, and poor in 14 patients (87.5%), one, and one, respectively. The mean PMA score was 16.1 (range 13–18) and the mean VAS score was 1.0 (range 0–3). No secondary reduction loss or implant loosening was observed. However, 2 patients underwent conversion to total hip arthroplasty (THA) due to post-traumatic arthritis and subsequent joint pain. No other complications were observed. In the comparative analysis, radiological outcome showed a significant relationship with the conversion to THA (*p* = 0.013). Shorter operation time and less blood loss were significantly observed in the QLS plate fixation group through the modified Stoppa approach compared with the conventional reconstruction plate fixation group through modified ilioinguinal approach (*p* < 0.001, respectively). Simultaneous reduction and fixation using an anatomical suprapectineal QLS plate through the modified Stoppa approach may be a viable technique in superomedially displaced acetabular fractures along with shorter operation time and less blood loss.

## Introduction

Superomedially displaced acetabular fractures generally involve two main column fragments. Although they are not specifically classified under a single type according to the established classification systems, these fractures involve both a superiorly displaced anterior column fragment and a medially displaced posterior column fragment including a quadrilateral surface (QLS)^1,2^. The treatment of these fractures is challenging because of the two-directionally displaced column fragments and their proximity to neurovascular structures^3,4^. Therefore, the use of appropriate surgical approach and reduction methods is very important for achieving satisfactory outcomes in patients with these fractures.

Traditional ilioinguinal and/or Kocher-Langenbeck approaches do not provide a sufficient surgical field for the medially displaced QLS fragment in these fractures; therefore, anatomical reduction and secure fixation of these fractures using these approaches is not easy^5–9^. Meanwhile, the modified Stoppa approach offers direct access to the medial aspect of the posterior column, including a QLS from the ischial spine to the greater sciatic notch^10^. Thus, this approach can facilitate the acquisition of adequate reduction and fixation in superomedially displaced acetabular fractures, even with fracture extension into the iliac wing and sacroiliac joint.

**Figure 1:**
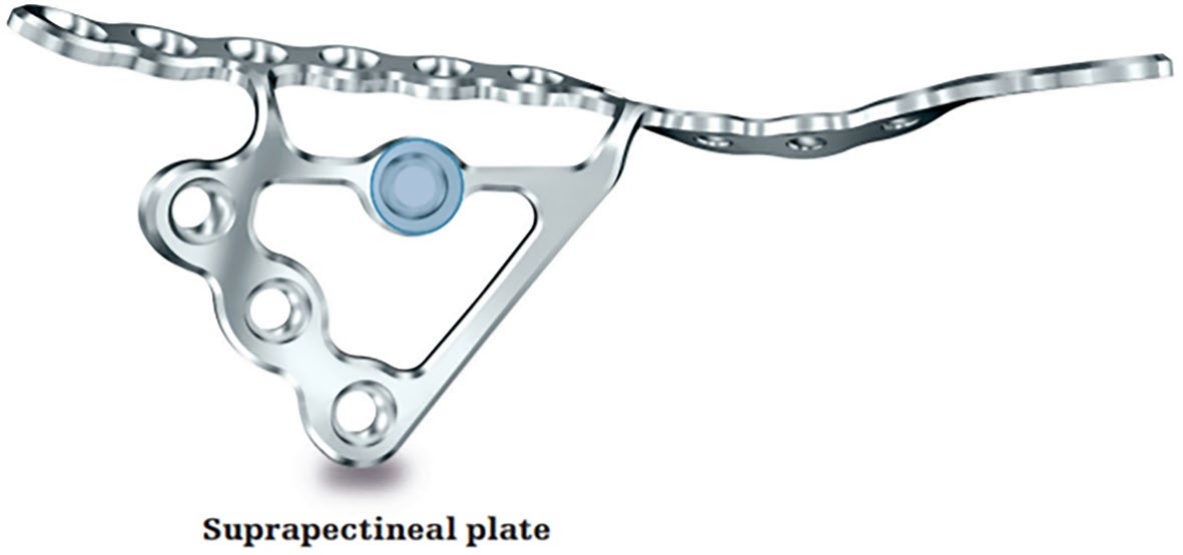
Image of anatomical suprapectineal quadrilateral surface plate.

The choice of an appropriate reduction method and tool is as important as the appropriate approach for the treatment of superomedially displaced acetabular fractures^11,12^. Several reduction methods using various clamps and reduction forceps have been used to treat these complex fractures. However, because of the displacement of the two column components into two separate directions and their location in the true pelvis, satisfactory reduction is difficult to achieve in these fractures. Furthermore, plate-and-screw fixation may be difficult due to the small space under the reduction devices, and the subsequent delay in fixation may lead to prolonged operation times and more bleeding^13–15^. An anatomical suprapectineal QLS plate (Stryker®, Selzach, Switzerland) was recently introduced and has been widely used in complex acetabular fractures (Fig. 1). This plate may also be a good option for the treatment of superomedially displaced acetabular fractures. We have performed reduction and fixation simultaneously using this plate via the modified Stoppa approach in these two-directionally displaced fractures and could reduce operation time and bleeding while yielding favorable outcomes.

This study aimed to report our surgical technique using an anatomical suprapectineal QLS plate through the modified Stoppa approach in superomedially displaced acetabular fractures and to demonstrate the viability of this plate along with the favorable surgical outcomes. Our hypothesis is that simultaneous reduction and fixation using an anatomical suprapectineal QLS plate through the modified Stoppa approach for superomedially displaced acetabular fractures yields comparable outcomes to the conventional fixation method using a reconstruction plate through modified ilioinguinal approach, along with shorter operation time and less blood loss than that.

## Materials and methods

This study was approved by the institutional review board of Hallym University Sacred Heart Hospital (2021-04-007). The institutional review board waived the informed consent for this study owing to its retrospective nature. All methods were carried out in accordance with the relevant guidelines and regulations.

Between June 2018 and June 2020, 20 superomedially displaced acetabular fractures were treated at our institute by using an anatomical suprapectineal QLS plate through the modified Stoppa approach. None of our patients received conservative treatment for this fracture type during the same period. Of the 20 patients, two had periprosthetic acetabular fractures, one was lost to follow-up, and one died 3 months after the operation due to causes unrelated to the index surgery. Finally, 16 patients (14 men, 2 women) with the mean age of 61.2 years (range 46–86 years) were enrolled in the current study.

Three-dimensional computed tomography (3D-CT) was performed in all patients before surgery to ensure accurate assessment of fracture pattern and obtain a detailed preoperative plan. The specific fracture pattern of superomedially displaced fractures, which included a superiorly displaced anterior-column component and a medially displaced posterior-column component involving a QLS, was confirmed on preoperative radiographs and 3D- CT. On the basis of the Letournel and Judet classification, 11 patients had both-column fractures while 5 patients had anterior-column and posterior hemitransverse fractures (Table 1). The average time from injury to surgery was 4.2 days (range 2–8 days).

**Table 1:** Data for 16 patients using a quadrilateral surface plate through modified Stoppa approach.

Case	Gender	Age	Fracture type	Accompanying fracture	Injury mechanism	Associated injuries
1	Male	46	BC	Posterior wall	Fall more than 3 m	Traumatic pneumothorax
2	Male	80	ACPH		Fall less than 3 m	
3	Male	52	BC		Fall less than 3 m	Ipsilateral pisiform fracture
4	Male	46	BC		Fall more than 3 m	Lumbar compression fracture
5	Male	53	ACPH	Anterior wall	Motorcycle road traffic accident	
6	Male	86	BC		Fall less than 3 m	
7	Male	52	BC		Fall more than 3 m	Contralateral scapular fracture
8	Female	67	BC	Anterior wall	Fall less than 3 m	Ipsilateral distal radius fracture
9	Male	54	BC	Anterior wall	Fall less than 3 m	Skull base fracture
10	Male	58	BC		Fall less than 3 m	
11	Female	80	BC	Anterior wall	Fall less than 3 m	
12	Male	67	ACPH	Anterior wall	Pedestrian road traffic accident	
13	Male	70	BC	Posterior wall	Crush by heavy objects	Multiple compression fractures
14	Male	61	ACPH		Bicycle road traffic accident	
15	Male	46	ACPH		Fall less than 3 m	
16	Male	62	BC		Fall less than 3 m	

*BC* both column fracture, *ACPH* anterior column and posterior hemitransverse fracture.

For comparative analysis, we collected the demographic and radiological data of 23 patients for superomedially displaced acetabular fractures involving a QLS fixed with the conventional reconstruction plate and buttress plate using a T-plate or reconstruction plate through modified ilioinguinal approach between February 2010 and May 2018. Because we have performed only the modified Stoppa approach since using an anatomical QLS plate in these fracture types, we set the conventional reconstruction plate fixation group through modified ilioinguinal approach as a control group for the comparative analysis. This group was composed of 20 men and 3 women with the mean age of 53.5 years (range 33–78 years) (Table 2). 18 patients had both-column fractures while 5 patients had anterior-column and posterior hemitransverse fractures. The average time from injury to surgery was 5.8 days (range 2–12 days).

**Table 2:** Data for 23 patients using a conventional reconstruction plate through modified ilioinguinal approach.

Case	Gender	Age	Fracture type	Accompanying fracture	Injury mechanism	Associated injuries
1	Male	39	ACPH	Anterior and posterior wall	In car traffic accident	Pneumocephalus
2	Male	43	BC		In car traffic accident	Ipsilateral olecranon fracture
3	Male	41	BC	Posterior wall	Motorcycle road traffic accident	Cerebral concussion
4	Female	54	BC	Posterior wall	Fall more than 3 m	
5	Female	36	ACPH	Anterior wall	In car traffic accident	
6	Male	57	BC		Fall less than 3 m	
7	Male	38	BC	Anterior wall	In car traffic accident	Hemoperitoneum
8	Male	62	ACPH	Anterior wall	Fall more than 3 m	Hemopneumothorax,
9	Male	57	ACPH		Fall less than 3 m	
10	Male	63	BC		Fall more than 3 m	Retroperitoneal hematoma
11	Male	59	ACPH		Fall more than 3 m	
12	Male	64	BC		Pedestrian road traffic accident	Multiple compression fractures
13	Male	65	BC	Anterior wall	Fall less than 3 m	
14	Male	55	BC		Fall less than 3 m	
15	Male	61	BC	Anterior and posterior wall	Fall less than 3 m	Ipsilateral distal radius fracture
16	Male	49	BC		Fall more than 3 m	Ipsilateral distal radius fracture
17	Male	66	BC		Fall less than 3 m	
18	Male	42	BC		In car traffic accident	Bilateral pneumothorax
19	Male	52	BC	Anterior wall	Fall less than 3 m	
20	Female	33	BC		Fall more than 3 m	Retroperitoneal hematoma
21	Male	47	BC	Anterior wall	Fall less than 3 m	
22	Male	78	BC	Anterior wall	Fall less than 3 m	
23	Male	48	BC		Fall less than 3 m	

*BC* both column fracture, *ACPH* anterior column and posterior hemitransverse fracture.

### Surgical techniques and procedures

**Figure 2:**
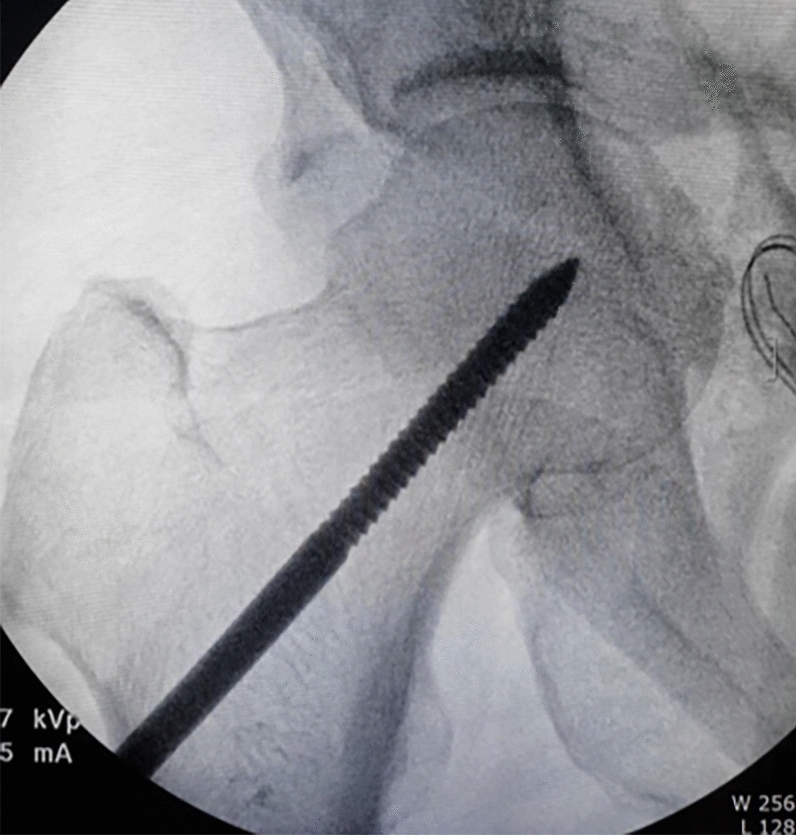
A fluoroscopic image of the insertion of the Schanz screw into the femoral head on operating side.

**Figure 3:**
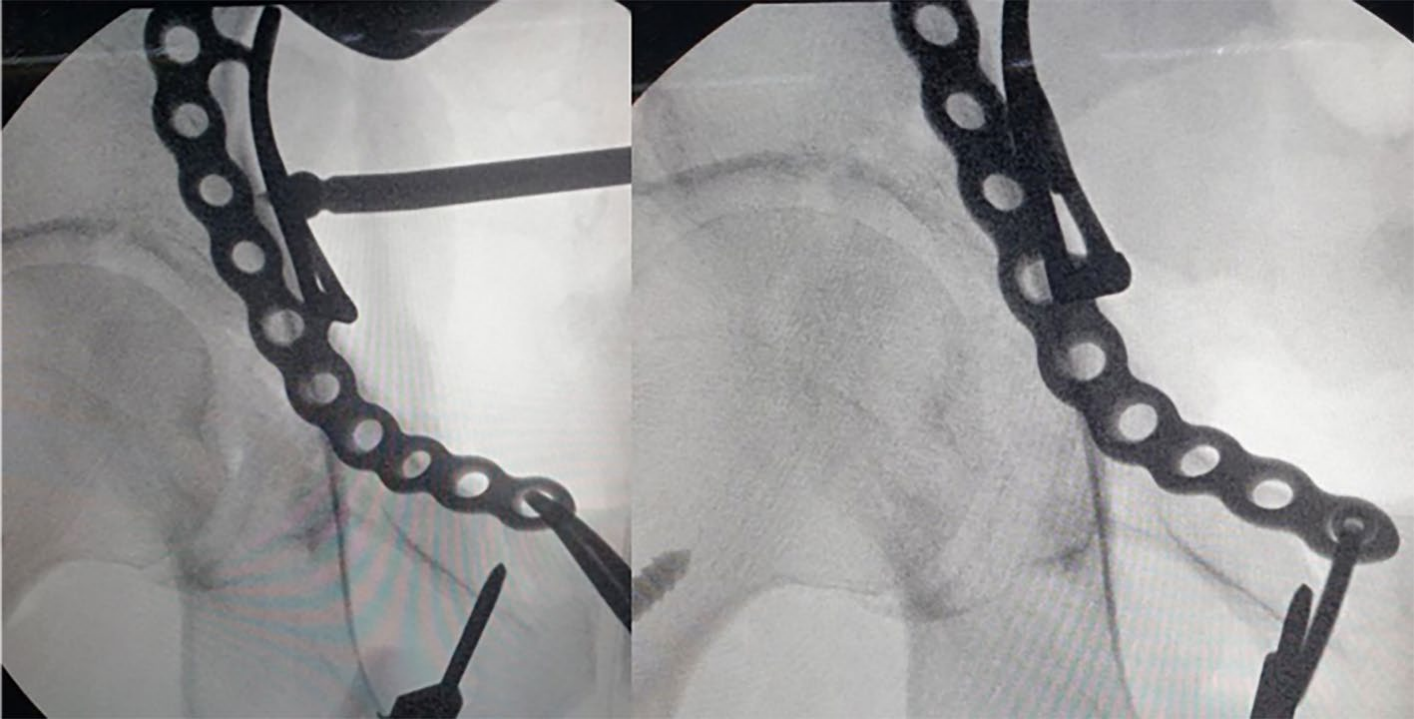
Fluoroscopic images of attaching the quadrilateral surface plate to quadrilateral surface using a ball spike pusher.

**Figure 4:**
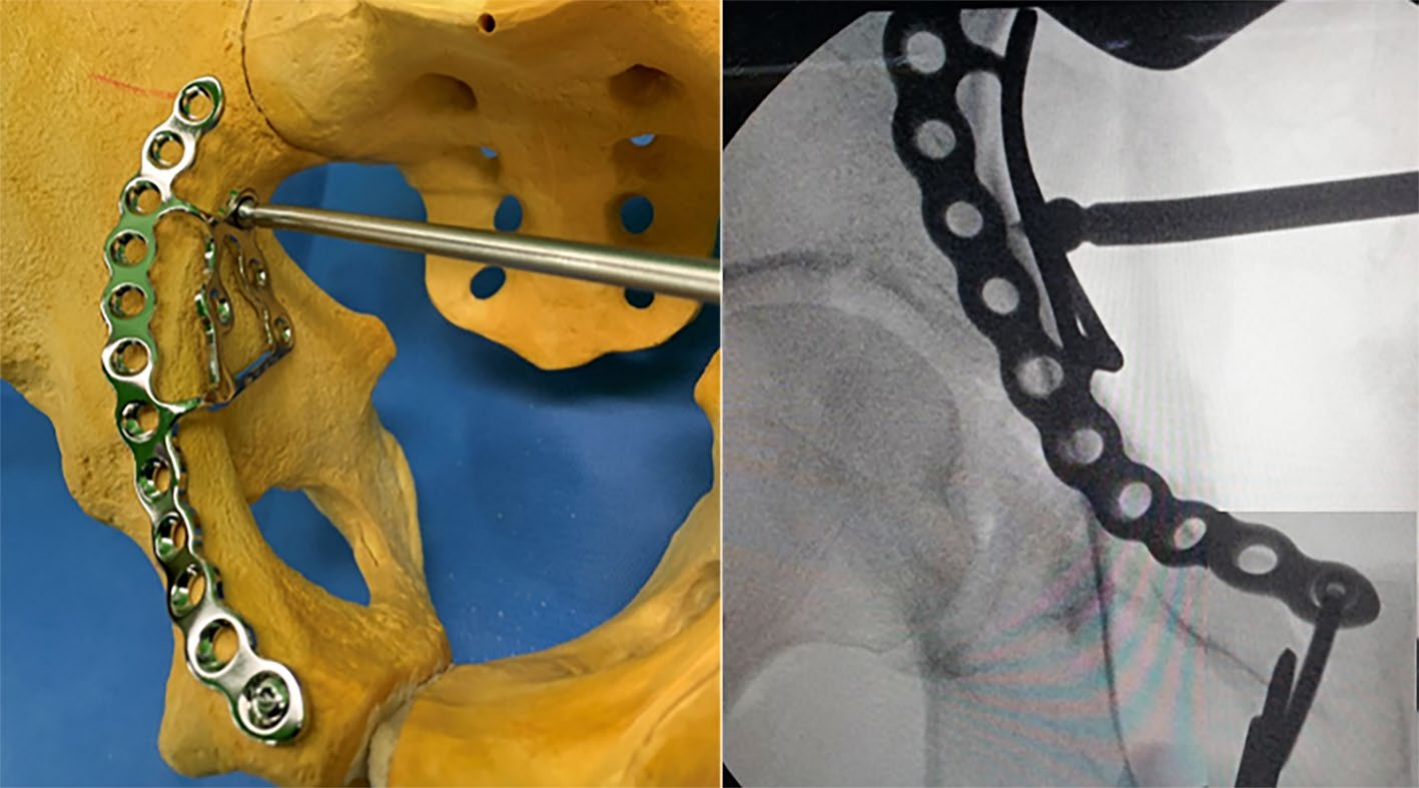
Indirect reduction of the medially displaced posterior column fragment using a ball spike pusher on the infrapectineal portion of the quadrilateral surface plate.

**Figure 5:**
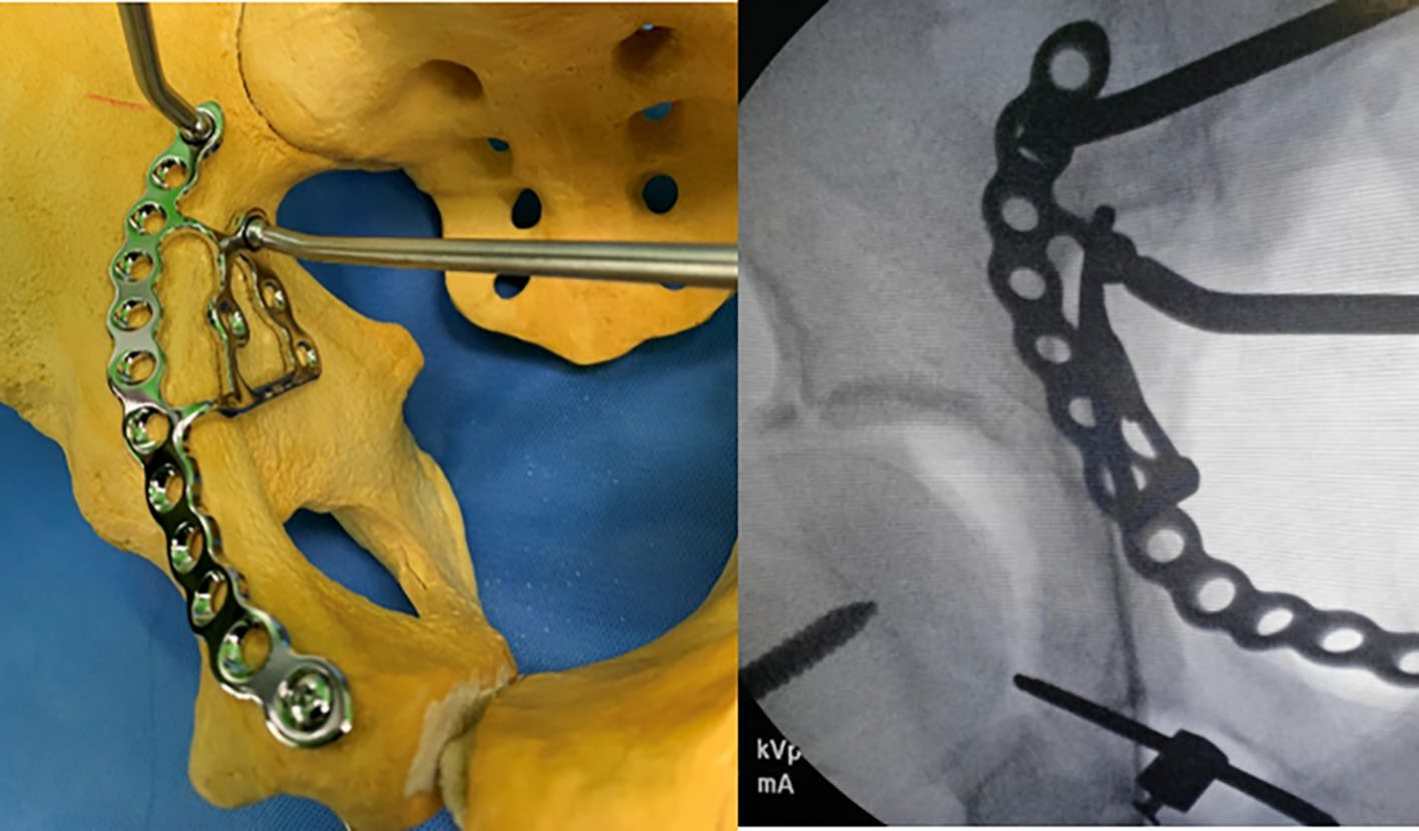
Reduction of the superiorly displaced anterior column using a pusher on the suprapectineal portion of the plate.

**Figure 6:**
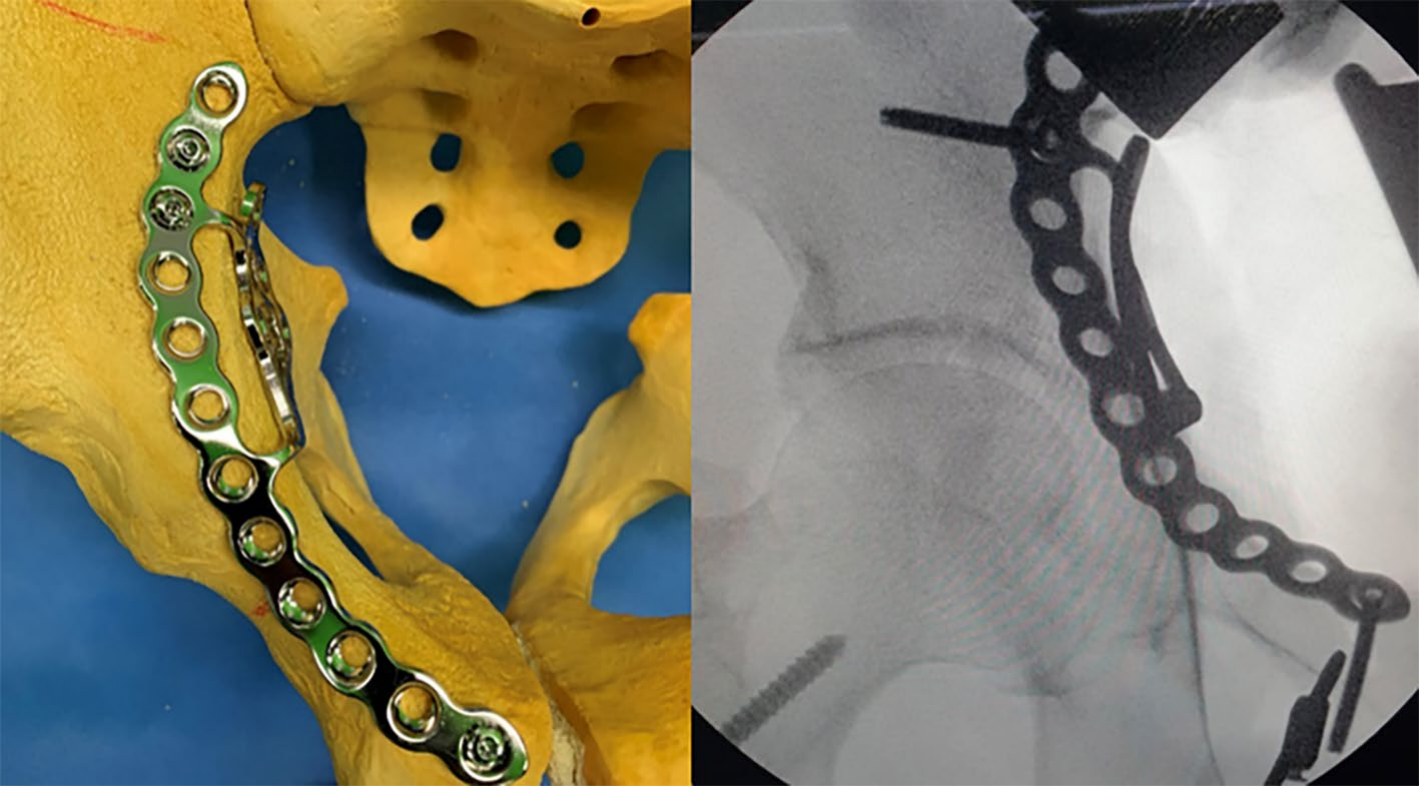
Insertion of the screw in the third hole from the back of a suprapectineal portion of the plate while the pushers were maintained on the plate.

All operations were performed by one hip and trauma surgeon (JHY) with an experience of > 10 years in pelvic-acetabular fractures. Surgery was performed with the patient in the supine position on a radiolucent table and with the ipsilateral hip in a flexed position, which was maintained throughout the procedure to relax the tension of the femoral neurovascular bundle. The modified Stoppa approach, as described by Cole and Bolhofner^10^, was used in all patients. In cases with complete iliac wing fractures (case 1 and 6), direct reduction and fixation with a 3.5 mm reconstruction plate or cortical screws was first performed through the additional lateral window. Next, the insertion of the rectus in the ipsilateral anterior pubic body was partially released to reduce the tension of the muscle and obtain sufficient visualization. To reduce the medially displaced QLS component more easily, the Schanz screw was inserted into the femoral head for lateral traction (Fig. 2). Subsequently, separation of soft tissues including the obturator vessels and muscles from the pelvic brim and QLS, was subperiosteally performed with a periosteal elevator. After obtaining a sufficient surgical field, an anatomical QLS plate was placed appropriately on the pelvic brim under fluoroscopic guidance so that its medial margin was close to the symphysis pubis while attaching the plate to the QLS using a ball-spike pusher, and the frontmost screw was inserted to fix the plate to the pubic body (Fig. 3). Next, indirect reduction of the medially displaced posterior column fragment including a QLS was achieved by pushing it laterally with a ball-spike pusher on the infrapectineal portion of the plate along with lateral traction of the femoral head using a Schanz screw (Fig. 4). While maintaining the reduction of the posterior column fragment, the superiorly displaced anterior column was reduced by pushing it inferiorly with another pusher on the suprapectineal portion of the plate (Fig. 5). After confirming satisfactory reduction of the anterior and posterior columns under direct visualization and fluoroscopic guidance, one screw was inserted toward the posterior column in the third hole from the back of the suprapectineal portion of the plate while the pushers were maintained on the plate for reduced anterior and posterior column fragments (Fig. 6). Depending on the fracture pattern and bone quality, additional screws were inserted in the anterior and posterior holes of the suprapectineal portion of the QLS plate: poorer-quality bone necessitated the insertion of more screws for secure fixation. Finally, simultaneous indirect reduction and fixation of superomedially displaced acetabular fractures using this plate was achieved, which was followed by compression between the anterior and posterior column components by screws inserted toward the posterior column. In patients (case 8, 9, 11, and 12) with a relatively large anterior wall fragment or comminution, a 3.5 mm reconstruction plate was additionally inserted as a buttress plate on the anterior wall right outside the QLS plate. Meanwhile, in patients (case 1 and 13) with an unreduced large posterior wall fragment through the anterior approach and fixation, additional fixation was performed using the Kocher-Langenbeck approach 1 week after anterior fixation. Correct reduction and implant positioning were carefully confirmed using fluoroscopy prior to wound closure.

After surgery, non-weight-bearing on the operated side was maintained for approximately 4 weeks; thereafter, tolerable weight-bearing with a pair of crutches was allowed. Full weight-bearing was permitted approximately 10 weeks after surgery, depending on the degree of radiographic fracture consolidation. Throughout the postoperative period, isometric quadriceps contraction exercises with the leg in extension were encouraged.

### Postoperative assessments

The quality of reduction of the articular surface and the congruency of the hip joint were evaluated on postoperative plain radiographs by using the Matta classification system, and the findings were categorized as anatomic (0–1 mm), imperfect (1–3 mm), and poor (> 3 mm)^11^.

At the final follow-up, the Postel Merle d’Aubigne (PMA) score and visual analog scale (VAS) score were used to rate the final clinical outcome. The radiological evaluation performed on the basis of the Matta criteria was as follows^11^: excellent (normal-appearing hip joint), good (mild changes with minimal sclerosis and joint narrowing < 1 mm), fair (intermediate changes with moderate sclerosis and joint narrowing < 50%), and poor (advanced changes). During the follow-up period, complications such as reduction loss, infection, nerve palsy, post-traumatic arthritis, venous thromboembolism, and heterotopic ossification were investigated. We defined the patients with secondary reduction loss, post-traumatic arthritis, and conversion to total hip arthroplasty (THA) as the failure group.

### Statistical analyses

Basic descriptive statistical analyses were used to describe the study population. For comparison between two groups (union group vs. failure group, QLS plate group vs. conventional reconstruction plate group), Student’s *t*-test was used for continuous variables. For categorical variables, the chi-square test was used, whereas Fisher’s exact test was used when the expected counts were < 5. *p*-value < 0.05 was considered statistically significant. All values were obtained using the statistical package SPSS version 17.0 (SPSS Inc., Chicago, IL, USA).

## Results

All patients were followed up for a minimum of 1 year with a mean follow-up period of 24.1 months (range 12–44 months), (Table 3). Assessments using plain radiographs showed that anatomical reduction of the acetabular fracture was achieved in 14 patients (87.5%), imperfect reduction was achieved in one and poor in one.

**Table 3:** Surgical outcomes of 16 patients using a quadrilateral surface plate through modified Stoppa approach.

Case	Follow-up period (month)	Surgical approach	Additional fixation	VAS score	PMA score	Time to operation (day)	Operation time (min)	Intraoperative bleeding	Time to union (month)	Quality of reduction	Radiographic grade	Complication
1	33	MS and KL	Iliac crest and posterior wall plate	1	Good (17)	3	155	1000	4	Anatomical	Excellent	
2	32	MS		2	Good (15)	4	145	1000	4	Anatomical	Excellent	
3	44	MS		0	Excellent (18)	2	90	500	3	Anatomical	Excellent	
4	33	MS		0	Good (17)	5	105	900	3	Anatomical	Excellent	
5	37	MS		0	Excellent (18)	2	60	750	4	Imperfect	Poor	THA conversion
6	27	MS	Iliac crest plate	1	Good (16)	3	75	1000	3	Anatomical	Excellent	
7	24	MS		0	Excellent (18)	7	70	800	3	Anatomical	Excellent	
8	25	MS	Anterior wall screw fixation	3	Fair (13)	2	85	500	4	Poor	Fair	THA conversion
9	24	MS	Anterior wall screw fixation	0	Good (17)	8	120	800	4	Anatomical	Excellent	
10	24	MS		0	Excellent (18)	4	115	1500	3	Anatomical	Excellent	
11	15	MS	Anterior wall screw fixation	1	Fair (14)	5	75	300	4	Anatomical	Excellent	
12	17	MS	Anterior wall buttress plate	2	Fair (14)	4	135	800	3	Anatomical	Excellent	
13	12	MS and KL	Posterior wall plate	0	Good (17)	6	180	1500	4	Anatomical	Excellent	
14	14	MS		2	Good (16)	4	100	500	3	Anatomical	Excellent	
15	13	MS		1	Good (15)	4	115	800	3	Anatomical	Excellent	
16	12	MS		2	Good (15)	3	130	1000	4	Anatomical	Excellent	

*M-S* Modified Stoppa, *K-L* Kocher-Langenbeck, *VAS* visual analogue scale, *PMA* Postel Merle d’Aubigné, *THA* total hip arthroplasty.

At the final follow-up, radiographic grades were excellent in 14 patients (87.5%), fair in one, and poor in one. The mean PMA score was 16.1 (range 13–18); evaluated excellent in 4 cases (25.0%), good in 9 (56.3%), and fair in 3 (18.7%), while the mean VAS score was 1.0 (range 0–3).

Postoperative follow-up radiographs showed no secondary reduction loss or implant loosening. However, mild protrusion of the femoral head along with joint space narrowing were observed in 2 patients (12.5%) who eventually underwent conversion to THA due to post-traumatic osteoarthritis and subsequent joint pain. Complications such as deep infection, surgical site infection, venous thromboembolism, or heterotopic ossification were not reported. Moreover, none of the patients showed iatrogenic lesions in the obturator neurovascular bundle. All patients could ambulate without external support at the last follow-up. Surgical outcomes of the conventional reconstruction plate group are shown in the Table 4. The traumatic osteoarthritis with joint space narrowing was observed in 2 patients (8.7%), who did not undergo conversion to THA, and lumbosacral plexopathy was observed in one.

**Table 4:** Surgical outcomes of 23 patients using a conventional reconstruction plate through modified ilioinguinal approach.

Case	Follow-up period (month)	Surgical approach	Additional fixation	VAS score	PMA score	Time to operation (day)	Operation time (min)	Intraoperative bleeding	Time to union (month)	Quality of reduction	Radiographic grade	Complication
1	78	II and KL	Anterior and posterior wall plate, and iliac crest plate	0	Excellent (18)	12	120	2800	4	Anatomical	Excellent	
2	93	II	Iliac crest plate	1	Excellent (18)	10	305	2000	6	Anatomical	Excellent	
3	31	II and KL	Posterior wall plate	0	Excellent (18)	5	220	1600	3	Anatomical	Excellent	
4	120	II and KL	Posterior wall plate	0	Good (17)	6	265	1000	3	Anatomical	Excellent	
5	116	II	Iliac crest plate and anterior wall screw fixation	0	Good (16)	2	235	2000	4	Anatomical	Excellent	
6	84	II	Iliac crest plate	1	Good (17)	6	205	1500	3	Anatomical	Excellent	
7	102	II	Iliac crest plate and anterior wall screw fixation	0	Good (17)	9	325	3000	8	Anatomical	Excellent	Lumbosacral plexopathy
8	24	II	Iliac crest plate and anterior wall screw fixation	1	Excellent (18)	10	300	800	3	Anatomical	Excellent	
9	54	II		1	Excellent (18)	2	165	1200	4	Anatomical	Excellent	
10	74	II		1	Good (17)	4	220	3000	4	Anatomical	Excellent	
11	73	II	Iliac crest plate	0	Good (17)	3	190	2000	5	Anatomical	Excellent	
12	23	II		0	Good (16)	7	385	1200	3	Anatomical	Excellent	
13	102	II	Anterior wall screw fixation	2	Fair (14)	7	180	2000	4	Imperfect	Good	Joint space narrowing
14	34	II	Iliac crest plate	0	Good (16)	3	180	1000	4	Anatomical	Excellent	
15	77	II and KL	Anterior wall buttress and posterior wall plate	2	Good (15)	6	115	3200	4	Imperfect	Good	Joint space narrowing
16	36	II		0	Good (17)	5	220	2000	3	Anatomical	Excellent	
17	24	II		0	Good (17)	2	300	1000	4	Anatomical	Excellent	
18	12	II	Iliac crest plate	0	Excellent (18)	9	285	2500	3	Anatomical	Excellent	
19	15	II	Anterior wall buttress plate	1	Good (17)	3	210	1000	6	Anatomical	Excellent	
20	58	II		0	Excellent (18)	9	535	1100	3	Anatomical	Excellent	
21	12	II		2	Excellent (18)	3	145	2500	4	Anatomical	Excellent	
22	26	II		1	Good (17)	3	85	1500	5	Anatomical	Excellent	
23	53	II	Iliac crest plate	0	Excellent (18)	7	240	2500	3	Anatomical	Excellent	

*I-I* Ilioinguinal, *K-L* Kocher-Langenbeck, *VAS* visual analogue scale, *PMA* Postel Merle d’Aubigné.

In the comparative analysis between the union group and failure group, we observed a significant relationship between the conversion to THA and the radiological outcome (*p* = 0.013). However, no significant differences were observed in the age, time to operation, operation time, and intraoperative bleeding. Meanwhile, shorter operation time and less intraoperative blood loss were significantly observed in the QLS plate fixation group through the modified Stoppa approach compared with the conventional reconstruction plate fixation group through modified ilioinguinal approach group (*p* < 0.001, respectively), (Table 5).

**Table 5:** Comparison between the QLS plate and conventional reconstruction plate groups.

	QLS plate group (n = 16)	Conventional reconstruction plate group (n = 23)	*p*-value
Age (years)	61.2 ± 12.8	53.5 ± 11.6	0.132
Time to operation (days)	4.2 ± 1.7	5.8 ± 3.0	0.114
Operation time (min)	109.9 ± 33.6	236.1 ± 97.9	< 0.001
Intraoperative bleeding (ml)	853.1 ± 329.3	1843.5 ± 752.5	< 0.001
Quality of reduction	0	0	0.875
Anatomical	14 (87.5%)	21 (91.3%)	
Imperfect	1 (6.2%)	2 (8.7%)	
Poor	1 (6.2%)	0	
Radiographic grade			0.875
Excellent	14 (87.5%)	21 (91.3%)	
Good	1 (6.2%)	2 (8.7%)	
Fair	1 (6.2%)	0	

QLS: quadrilateral surface, Continuous variables are presented as mean ± standard deviation.

## Discussion

Superomedially displaced acetabular fractures are generally caused by the medial impact of the femoral head into the QLS and superior dome, which displaces the anterior column superiorly and the posterior column including the QLS medially^1^. These acetabular fractures involving a QLS are complex fractures that are not regarded as a parameter in the gold standard Judet-Letournel classification system. However, these fractures are mostly associated with anterior-column and posterior hemitransverse or both-column fractures, as shown in our study.

**Figure 7:**
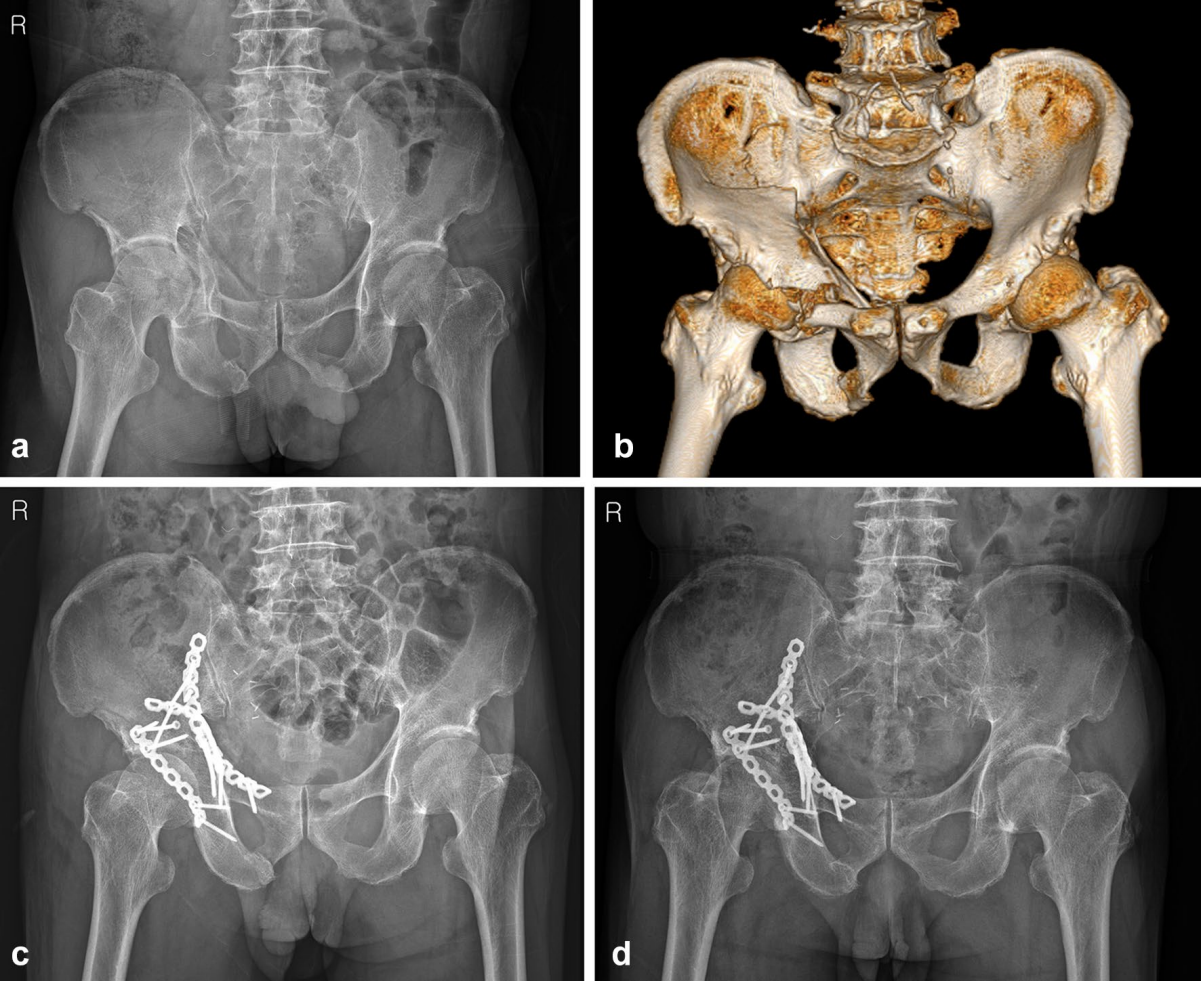
(**a**) pelvis anteroposterior radiograph and (**b**) 3D-CT of a 61-year-old male, superomedially displaced acetabular fracture with anterior wall fragment. (**c**) Immediate postoperative and (**d**) 77 months after surgery radiograph, after fixed with conventional reconstruction plate and additional buttress plate through modified ilioinguinal approach.

The appropriate approach and reduction method is of paramount importance for achieving satisfactory results in these two-directionally displaced fractures^9^. In the past, these fractures were fixed mainly with reconstruction plates through the ilioinguinal or combined approach with Kocher-Langenbeck approach^4,7^. However, conventional approaches cannot yield sufficient direct access to the posterior column component including a QLS, and secure stabilization is difficult to obtain using conventional fixation methods, especially for a medially displaced QLS fragment. Moreover, intraoperative reshaping of the conventional plates may be required to improve the buttress effect on the QLS fragment, which can prolong the operative time and reduce the buttress intensity of the plate (Fig. 7). Anterograde lag screws may be inserted from an anterior approach to fix the posterior column involving a QLS fragment, and an additional posterior incision may be unnecessary^15,16^. However, lag screws can reduce the stability of the fragments, especially in osteoporotic elderly patients or those with comminution of the pelvic brim or QLS, and the insertion technique is demanding^17,18^. As a result, complications including screw loosening and secondary reduction loss leading to protrusion of the femoral head may occur^9,19^.

**Figure 8:**
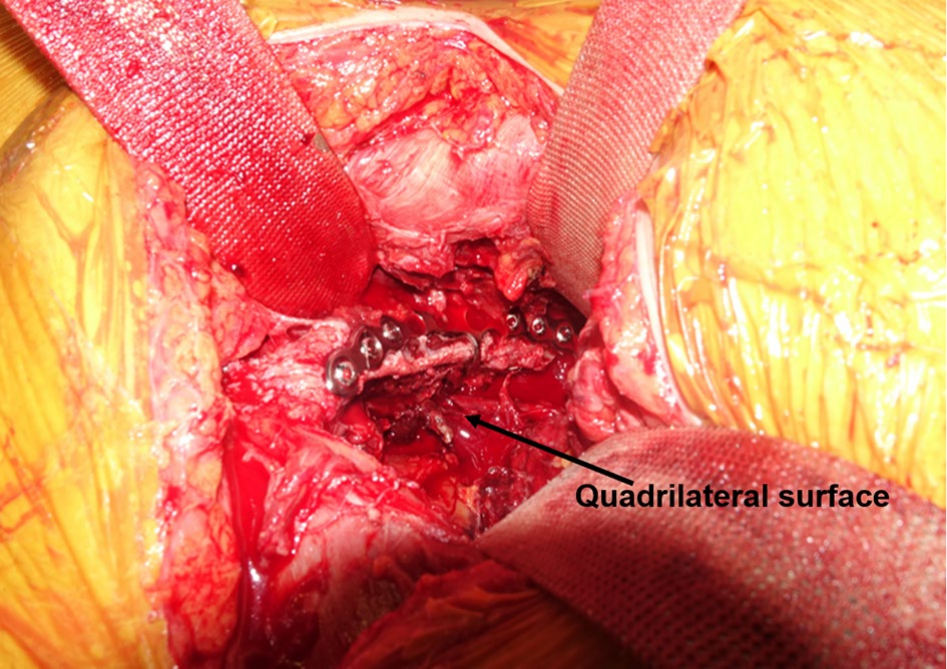
Intraoperative picture showing the excellent buttress effect of the plate for displaced quadrilateral surface.

Several authors have developed new fixation strategies for medial infrapectineal buttress plates in medially displaced QLS fractures^20,21^. However, this solution does not provide adequate support for superiorly displaced anterior-column fracture components in superomedially displaced acetabular fractures and can often hinder direct reduction of these components unless simultaneous reduction of the two-directionally displaced fracture components is performed. Meanwhile, as shown in our technique, the method using an anatomical suprapectineal QLS plate through the modified Stoppa approach can enable simultaneous indirect reduction and fixation of the posterior column component, including a QLS fragment along with the anterior column component. This anatomical QLS plate simultaneously serves as a reduction tool and fixation device in our technique; therefore, no additional reduction device or temporary fixation is necessary. This technique also avoids additional reshaping of the plate due to its anatomical contour, and sufficient stability of the QLS fragment can be achieved with this plate because of its excellent buttress effect (Fig. 8). We believe that the usefulness of our technique using the anatomical QLS plate comes from the characteristics of this plate. It is developed based on specific tools with massive database; a suite of tools which utilizes a comprehensive database of CT-scans, plus associated 3D bone models, allowing the user to assess population differences in bone morphology, bone density, and implant fit for the purposes of research and development^22^. Ciolli et al.^23^ also reported that this QLS plate was useful along with favorable outcomes in the treatment of different fracture types involving a QLS such as anterior column, anterior column-posterior hemitransverse, T-type, and both columns. We could also achieve satisfactory reduction and surgical outcomes along with shorter operation time and fewer complications in almost all cases using our technique. No fixation failures, such as screw loosening or secondary reduction loss leading to protrusion of the femoral head, were observed in the current study. Nevertheless, iatrogenic injuries of the obturator nerve and corona mortis may be accompanied during the placement of this plate because it is much larger than the conventional plate. However, these injuries can be avoided by timely ligation of the corona mortis and careful protection of the obturator nerve, as shown in our study.

In the literature, modified Stoppa approach has been reported to reduce the operation time and blood loss in the treatment of superomedially displaced acetabular fractures compared to ilioinguinal approach^3,14,24^. In contrast, some authors reported that there were no differences in the amount of bleeding or operation time between both approaches^25,26^. However, these studies were based on the fixation of the acetabular fractures using a conventional reconstruction plate. We believe that the result of the present study showing significantly shorter operation time and less blood loss is due to the synergic effect of our technique using the anatomical QLS plate and modified Stoppa approach^27^.

**Figure 9:**
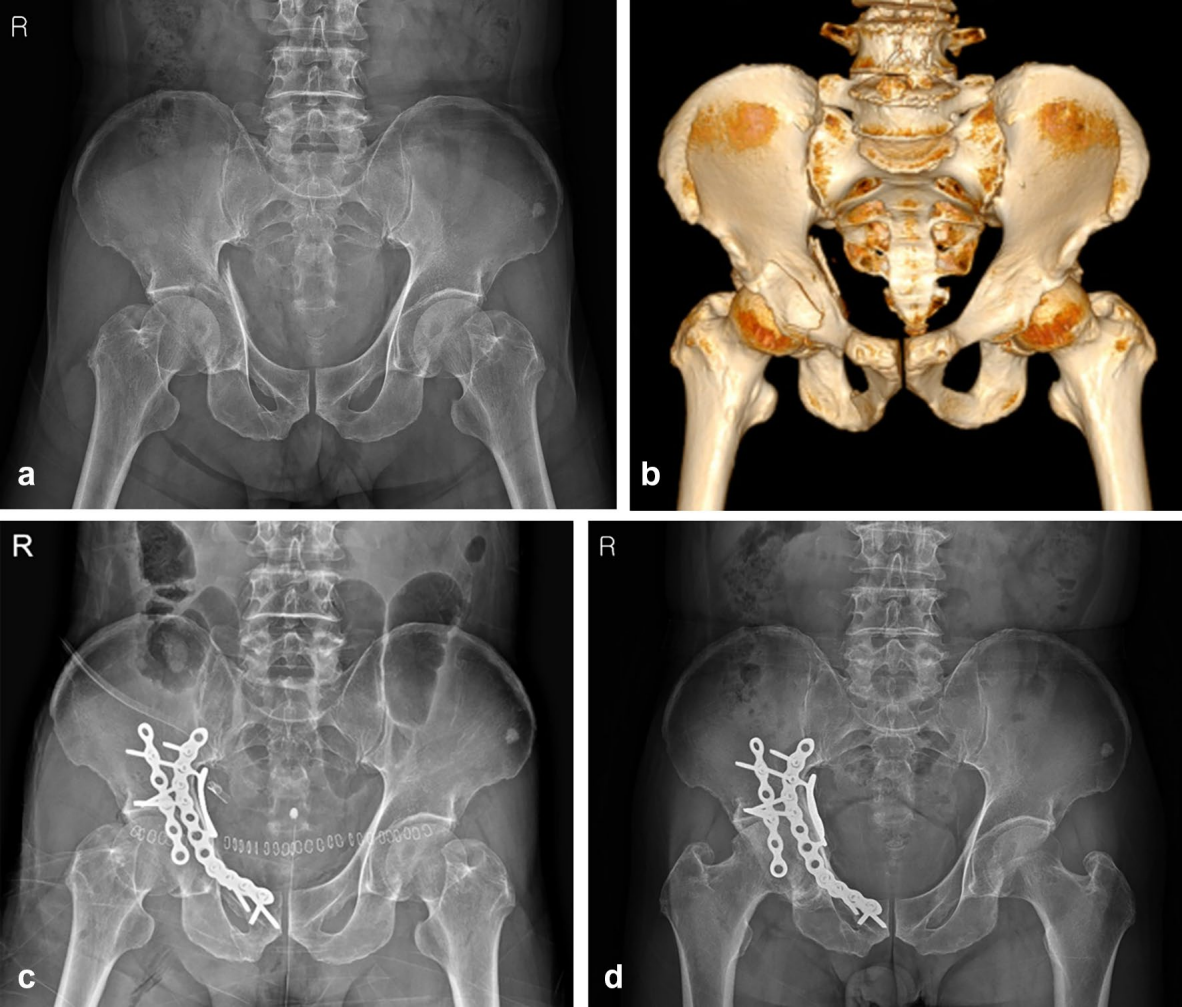
(**a**) pelvis anteroposterior radiograph and (**b**) 3D-CT of a 67-year-old male, superomedially displaced acetabular fracture with anterior wall fragment. (**c**) Immediate postoperative and (**d**) 18 months after surgery radiograph, after fixed with quadrilateral surface plate and additional buttress plate.

When indirect reduction of the medially displaced posterior column including a QLS is performed using this anatomical QLS plate and a ball-spike pusher according to our technique, the intact greater sciatic notch can serve as a reference mark for reduction of displaced posterior column fragment. However, in cases with an impacted dome fracture, which is often present concomitantly in elderly patients, reduction of the dome fracture using a Cobb’s elevator should be performed first through the fracture site between the anterior and posterior columns under direct visualization or fluoroscopic guidance. While maintaining the reduction of the posterior column component after its reduction, the superiorly displaced anterior column component is pressed using another ball-spike pusher on the plate; thus, compressive fixation between the anterior and posterior column components can be obtained by inserting screws toward the posterior column. In cases with relatively large unreduced anterior or posterior wall fragments, additional fixation should be performed for more anatomical reduction and firm fixation, as in our study. However, as shown in the current study, the 2 patients who underwent conversion to THA due to inadequate reduction and subsequent arthritis, showed a large fragment or comminution at the anterior wall on preoperative 3D-CT. In one patient with comminution of the anterior wall, no additional fixation was performed except for the anatomical QLS plate. Meanwhile, in the other patient with a large fragment of the anterior wall, two lag screws were inserted to fix this fragment additionally. Similar to posterior wall fracture, this anterior wall fracture affecting joint stability and congruency can also be considered as a poor prognostic factor. Accordingly, more careful attention is required for accurate reduction and firm fixation for this anterior wall fragment, and additional buttress plate is needed for this fragment to obtain more favorable outcomes (Fig. 9).

The present study had some limitations. The number of patients was relatively small and the follow-up period was relatively short. These factors may limit the clinical viability of our technique using the anatomical suprapectineal QLS plate. However, our simultaneous reduction and fixation technique using this plate through the modified Stoppa approach could serve as a minimally invasive treatment along with shorter operation time and less blood loss for superomedially displaced acetabular fractures with a QLS fragment.

We are not aware of any reduction and fixation techniques using an anatomical suprapectineal QLS plate and subsequent clinical series assessing the results treated with specific technique in superomedially displaced acetabular fractures with a QLS fragment. The current study demonstrates that superomedially displaced acetabular fractures can be treated successfully by simultaneous reduction and fixation technique using an anatomical suprapectineal QLS plate through the modified Stoppa approach along with favorable outcomes and fewer complications. Another strength of this study, it is the first study to compare the anatomical QLS plate fixation through the modified Stoppa approach and conventional reconstruction plate fixation through modified ilioinguinal approach.

In conclusion, superomedially displaced acetabular fractures involving a QLS can be treated more easily and effectively by our simultaneous reduction and fixation technique using the anatomical suprapectineal QLS plate through the modified Stoppa approach, leading to shorter operation time and less blood loss. However, lager cohort and long-term follow-up comparative studies are needed to confirm our results.

## Data Availability

Data is available from the corresponding author on reasonable request.
